# Extensive mitochondrial heteroplasmy in hybrid water frog (*Pelophylax* spp.) populations from Southeast Europe

**DOI:** 10.1002/ece3.1692

**Published:** 2015-09-28

**Authors:** Jelena M. Radojičić, Imre Krizmanić, Panagiotis Kasapidis, Eleftherios Zouros

**Affiliations:** ^1^ Hellenic Centre for Marine ResearchInstitute of Marine Biology, Biotechnology and AquacultureHeraklionGreece; ^2^ Department of BiologyUniversity of CreteHeraklionGreece; ^3^ Faculty of BiologyInstitute of ZoologyUniversity of BelgradeBelgradeSerbia

**Keywords:** Heteroplasmy, hybridization, microsatellites, mitochondrial DNA, *Pelophylax* (*Rana*) sp., water frogs

## Abstract

Water frogs of the genus *Pelophylax* (previous *Rana*) species have been much studied in Europe for their outstanding reproductive mechanism in which sympatric hybridization between genetically distinct parental species produces diverse genetic forms of viable hybrid animals. The most common hybrid is *P. esculentus* that carries the genomes of both parental species, *P. ridibundus* and *P. lessonae*, but usually transfers the whole genome of only one parent to its offsprings (hybridogenesis). The evolutionary cost of transfer of the intact genome and hence the hemiclonal reproduction is the depletion of heterozygosity in the hybrid populations. *Pelophylax esculentus* presents an excellent example of the long‐term sustained hybridization and hemiclonal reproduction in which the effects of the low genetic diversity are balanced through the novel mutations and periodic recombinations. In this study, we analyzed the mitochondrial (mt) and microsatellites DNA variations in hybrid *Pelophylax* populations from southern parts of the Pannonian Basin and a north–south transect of the Balkan Peninsula, which are home for a variety of *Pelophylax* genetic lineages. The mtDNA haplotypes found in this study corresponded to *P. ridibundus* and *P. epeiroticus* of the Balkan – Anatolian lineage (*ridibundus–bedriagae*) and to *P. lessonae* and a divergent *lessonae* haplotype of the *lessonae* lineage. The mtDNA genomes showed considerable intraspecific variation and geographic differentiation. The Balkan wide distributed *P. ridibundus* was found in all studied populations and its nuclear genome, along with either the *lessonae* or the endemic *epeiroticus* genome, in all hybrids. An unexpected finding was that the hybrid populations were invariably heteroplasmic, that is, they contained the mtDNA of both parental species. We discussed the possibility that such extensive heteroplasmy is a result of hybridization and it comes from regular leakage of the paternal mtDNA from a sperm of one species that fertilizes eggs of another. In this case, the mechanisms that protect the egg from heterospecific fertilization and further from the presence of sperm mtDNA could become compromised due to their differences and divergence at both, mitochondrial and nuclear DNA. The heteroplasmy once retained in the fertilized egg could be transmitted by hybrid backcrossing to the progeny and maintained in a population over generations. The role of interspecies and heteroplasmic hybrid animals due to their genomic diversity and better fitness compare to the parental species might be of the special importance in adaptations to miscellaneous and isolated environments at the Balkan Peninsula.

## Introduction

**Figure 1:**
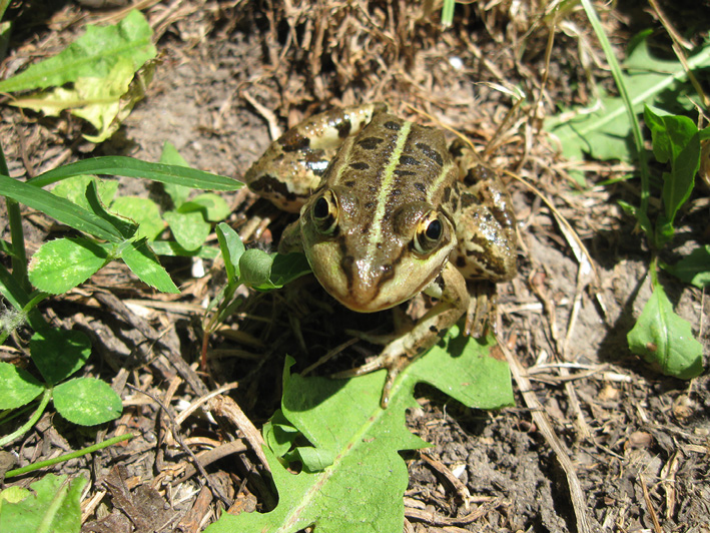
The marsh frog, *Pelophylax ridibundus*. Photograph: Imre Krizmanic

The western Palearctic water frogs of the genus *Pelophylax* have been classified into three phylogenetic lineages according to their mitochondrial DNA (mtDNA) variation: the *perezi* (*P. perezi* and *P. saharicus*), the *lessonae* (*P. lessonae, P. bergeri,* and *P. shqipericus*) and the most diverse *ridibundus*–*bedriagae* (*P. ridibundus*,* P. epeiroticus*,* P. cretensis*,* P. cypriensis*, and *P. bedriagae*) (Plotner 1998; Plotner and Ohst 2001; Lymberakis et al. 2007; Plotner et al. 2012). *Pelophylax lessonae* and *P. ridibundus* are extensively distributed in Europe, whereas the rest of water frog species are limited to its southern peninsulas. Complex geomorphology, climate history, and ecosystem diversity have resulted a remarkable divergence and endemism of both the *lessonae* and the *ridibundus*–*bedriagae* lineages at the Balkan Peninsula (Lymberakis et al. 2007; Plotner et al. 2010). *Pelophylax ridibundus* (Fig. 1) is abundant and widely distributed, while *P. shqipericus* in coastal Montenegro and northwestern Albania, *P. epeiroticus*, in western Greece, the island of Corfu and southern Albania, *P. cretensis* in the island of Crete and *P. cypriensis* in Cyprus have restricted distribution (Sofianidou and Schneider 1989; Beerli et al. 1996; Valakos et al. 2007; Plotner et al. 2012). A variety of *P. bedriagae* genetic lineages are found in eastern parts of Thrace in Greece, the east Aegean islands, in eastern Mediterranean, and the Middle East (Akin et al. 2010; Plotner et al. 2010, 2012). Combination of the biogeographic history and present biodiversity of the Balkan Peninsula and eastern Mediterranean makes these Pleistocene refugia exceptional for studying phylogeography and process of speciation (Schmitt 2007).

*Pelophylax ridibundus* and *P. lessonae* are known to hybridize over much of their overlapping geographic distribution in Europe and their hybrids, known as *P. esculentus*, form large populations that occur over a wide geographic area (Ragghianti et al. 2007). Numerous studies have shown that *P. esculentus* is a collective name for a number of genetically diverse forms, whose common characteristic is that they contain a combination of nuclear material from *P. ridibundus* and *P. lessonae* (Schmeller et al. 2005; Hotz et al. 2008). They can be diploid, containing a full haploid complement from each parental species, or triploids with two complements from one or the other species (Vinogradov et al. 1990; Ogielska et al. 2004; Christiansen et al. 2010). Diploid hybrids mate and reproduce with a sympatric parental species, while all‐hybrid triploid frog populations are reproductively independent from parents (Christiansen et al. 2010). *Pelophylax esculentus* is abundant in the northern part of the Balkan Peninsula, in which mostly diploid forms have been found (Spasic‐Boskovic et al. 1999; Krizmanic and Ivanovic 2010). This is in contrast to the considerable number of the pure triploid hybrid populations found in central and northern Europe (Plotner et al. 2008; Christiansen et al. 2010; Christiansen and Reyer 2011), or to coexisting diploid with triploid hybrids (Christiansen and Reyer 2009; Christiansen et al. 2010). *Pelophylax ridibundus* is also known to hybridize with other species found in the European Peninsulas, as *P. perezi* in the Iberian Peninsula (where the hybrid is named *P*. *grafi*), (Pagano et al. 2001), *P. bergeri* in Italy (Canestrelli and Nascetti 2008) and *P. epeiroticus* in western Greece and Peloponnese (Sofianidou and Schneider 1989; Sofianidou 1996).

The main form of reproduction in hybrids is hybridogenesis, a premeiotic process whose end result is the exclusion of the complete haploid set of chromosomes of one parental species from the mature egg or sperm (Tunner and Heppichtunner 1991; Ogielska and Bartmanska 1999; Archetti 2004; Marracci and Ragghianti 2008). Mature egg of a hybrid female is then fertilized by the sperm, which haploid genome could be from the same or different species from the one in the egg. The latter case reconstitutes the hybrid state in the progeny but also, due to the absence of recombination between parental genomes, accelerates the accumulation of deleterious mutations over generations (Guex et al. 2002). It has been suggested that new mutations and intermittent recombinations can improve genetic diversity in hemiclonal organisms and beneficially influence their fitness in small populations (Som and Reyer 2006, 2007).

A possible effect of hybridization between different species in nature is heteroplasmy, that is, the occurrence of two or more types of mtDNA in the same individual. A cause of heteroplasmy is somatic mutation in mtDNA that occurred in the individual in which the heteroplasmy was detected. The second cause is a “leakage” of the sperm's mtDNA into the egg during fertilization. This kind of heteroplasmy in which the portion of paternal mtDNA is a small minority in relation to the maternal mtDNA has been seen in insects, mussels, birds, and mammals, including humans (Kvist et al. 2003). In overwhelmingly homospecific crosses and maternal DNA inheritance in nature, the egg is provided with a cytoplasm factor that recognizes the label of the sperm mitochondria, which safeguards the new zygote from the presence of paternal mitochondria, either by the prevention of their entrance in the egg or by their destruction upon entering (Birky 2001). This mechanism works effectively in homospecific crosses in which the egg cytoplasm factor and the sperm label are encoded by the same nuclear background (Shitara et al. 1998). Nevertheless, this mechanism may become less effective in heterospecific crosses due to different nuclear backgrounds of species involved in the cross (Kaneda et al. 1995; Dokianakis and Ladoukakis 2014). As a result, the probability of the paternal mtDNA leakage into the new zygote may be higher the more diverged the two species involved in the cross become (Rokas et al. 2003).

In this study, we present the results from a survey of mtDNA and microsatellite variation in six *Pelophylax* spp. populations from southern parts of the Pannonian Basin and the Balkans, which include *P. ridibundus* and hybrids of *P. ridibundus* with either *P. lessonae* (*P. esculentus* forms in Serbia and Montenegro) or *P. epeiroticus* (Greek hybrids). We questioned the existence of mtDNA heteroplasmy in the studied populations and checked whether it would be more common in hybrids as opposed to parental species. For this purpose, we placed at first all mtDNA types from surveyed species and their hybrids within the wider phylogeny of the genus *Pelophylax* mitochondrial DNA that we knew to occur in the Balkan Peninsula. We checked how these variations are related to the previously characterized mtDNA types of *Pelophylax* spp. and how they are distributed among pure‐species and hybrids. Finally, from the mtDNA and microsatellite variation we inferred the degree of reproductive isolation among studied populations.

## Material and Methods

### Samples and DNA preparation

**Figure 2:**
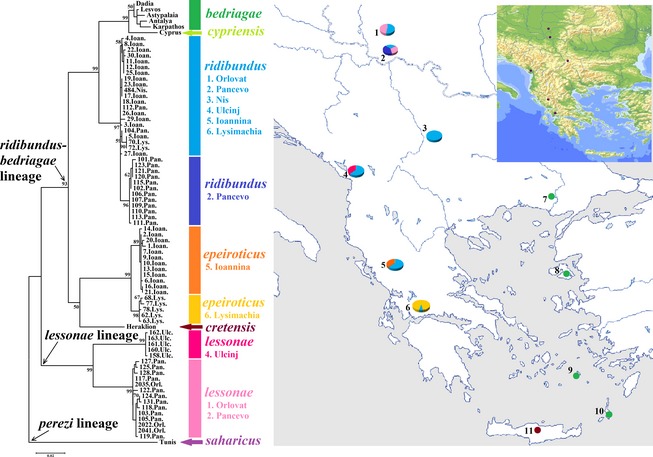
Maximum likelihood phylogeny of combined mtDNA genes, with distribution and frequency of the four mtDNA haplotypes (*rid*,* epe*,* les,* and *les* U) in the examined localities: 1. Orlovat, 2. Pancevo, 3. Nis, 4. Ulcinj, 5. Ioannina, and 6. Lysimachia. Additional haplotypes found in *Pelophylax bedriagae* are also presented: 7. Dadia, 8. Lesvos, 9. Astypalaia, 10. Karpathos, and 11. *Pelophylax cretensis* in Crete. GenBank accession codes: 7. DQ474215, DQ474163; 8. DQ474187, DQ474135; 9. DQ474192, DQ474140; 10. DQ474194, DQ474142; 11. DQ474199, DQ474147; *P. saharicus* AF215426.

*Pelophylax* specimens of this study originated from six different localities from southern parts of the Pannonian Basin and a north–south transect across the Balkan Peninsula. In total, 158 individuals were from three localities from Serbia, Orlovat, (23 individuals), Pancevo (31), and Nis (28), one from the Adriatic cost of Montenegro, Ulcinj, (13) and two from northern, Ioannina Lake (30) and central Greece, Lysimachia Lake (33) (Fig. 2). Samples belong to two herpetological collections (Department of Biology, University of Crete and Institute of Zoology, Faculty of Biology, University of Belgrade). Additional specimens from the herpetological collection of the Natural History Museum of Crete (NHMC) were also included in the analysis. They originate from northern and central Greece, Peloponnese, the islands Crete, Kithira, Karpathos, Lesvos, Chios, Astypalaia, and Cyprus, as listed in (Lymberakis et al. 2007). DNA was extracted from somatic tissue (tongue) using the salt extraction protocol (Miller et al. 1988). The quality of extracted total DNA was estimated by 1% agarose gel electrophoresis stained with ethidium bromide, in the presence of molecular size marker (Lambda DNA *Eco*RI/*Hin*d III digest ladder).

### Microsatellite markers

Microsatellite markers used in this study were chosen from the microsatellites genomic library previously developed for *P. lessonae* and *P. esculentus*, respectively. We used the following six loci: Res 5, Res 14, Res 16, Res 17, Res 20 (Zeisset et al. 2000), and RLCA1b5 (Garner et al. 2000) for genotyping 158 animals in this study (Table 1). The Res 5, Res 14, Res 16, and RlCA1b5 loci amplified the genomes of *P. ridibundus*,* P. lessonae,* and *P. epeiroticus* (herein being referred as *rid*,* les,* and *epe*, respectively). The locus Res 20 detected the *les* and *epe* genomes, while the Res17 the *rid* and *epe* ones. Additional specimens from Greece, 7 of *P. cretensis* from Crete and 11 of *P. bedriagae* from Karpathos, Chios, and Astypalaia, were also analyzed for the microsatellite variation and compared with animals from our study. Amplification of the microsatellite markers was carried out by PCR reaction of 10 *μ*L, which contained about 10 ng of total DNA, 1 *μ*L Taq buffer 10×, forward infrared‐labeled 0.8 *μ*mol/L primer, 0.5 *μ*mol/L reverse primer, 1.5–2 mmol/L MgCl_2_, 10 mmol/L dNTP's, 0.3 units/*μ*L of Taq DNA polymerase (Minotech, Heraklion, Crete, Greece) and nano‐pure sterilized water. Forward primer was modified with a heptamethine cyanine infrared dye (IRD 800). Template DNA was initially denatured at 94°C for 5 min, followed by 30 cycles of denaturation at 94°C for 45 sec, annealing temperature for 45 sec, extension at 72°C for 45 sec, and final 10 min extension at 72°C at the end. Microsatellite PCR products (80–250 bp size) were analyzed by electrophoresis through standard denatured sequencing gels (6% polyacrylamide gel) on the automated infrared fluorescent sequencer (Li‐COR 4200 Inc., Lincoln, NE, USA). The 6% acrylamide working solution was prepared in the presence of TBE buffer and urea. Polymerization of acrylamide was carried out by ammonium persulfate (APS) in the presence of tetramethylethylenediamine (TEMED). Microsatellite alleles were scored by size marker 50–350 bp (Li‐COR) and analyzed by Gene ImagIR software (2.3 version; Li‐COR Inc).

**Table 1:** Microsatellite allele variation in three species: *Pelophylax lessonae*,*Pelophylax ridibundus,* and *Pelophylax epeiroticus* in our study. *Pelophylax cretensis* and *Pelophylax bedriagae* are presented for allele variation comparison. Bold and underlined are private (species‐specific) alleles; dash denotes the absence of amplification product

Locus	Observed alleles	Size range	*P. lessonae a*lleles	*P. ridibundus* alleles	*P. epeiroticus* alleles	*P. cretensis* alleles	*P. bedriagae* alleles
1. Res 5	10	144–172	144, 146, 148, 150, 154, 156, 164, 166, **170**, **172**	144, 146	144, 146, 148, 150, 154, 164, 166	156, **158**, **160**	144, 148, **152**, 156
2. Res 14	6	132–148	138	132, 134, 140, 144, **148**	140, 144	132, 140, 144	**130**, 132, 134, 138, 140
3. Res 16	7	118–136	124	118, **120**, 122, 126, 130, 136	122, 124, 126	124, 130, **134**	**116**, 118, 124, 126, 130, **132**, 136, **150**
4. Res 17	8	150–168	–	150, 154, 156, 158, **160**, 166, **168**	150, **152**, 154, 156	150, 154, 158	150, 154, 166
5. Res 20	20	110–186	**110**, 112, **120**, **138**	–	112, **116**, **118**, **130**, **134**, **142**, **146**, **150**, **154**, **158**, **160**, **162**, **166**, **170**, **178**, **182**, **186**	–	–
6. RlCA1b5	11	120–144	**120**, **122**, 126	130, 132, 134, 136, 138, **140**, **142**, **144**	126, 134	126, **128**	**124**, 126, 130, 132, 134, 136, 138, **158**
Total number of alleles	62	–	19	28	35	14	28
Number of private alleles	42	–	7	7	17	4	7

### Amplification of two mtDNA genes and direct PCR product sequencing

Two mitochondrial DNA regions were targeted: the large subunit ribosomal RNA gene (16S rRNA) and the cytochrome *b* (cyt *b*). We used the universal 16S rRNA primers (Palumbi 1996) that amplify a fragment of 500–650 bp from the 16S rRNA gene of most animal taxa. For the cyt *b* gene, we used originally the primers given by (Tanaka et al. 1996), which work in most frog species. Information about the pairs of primers used and amplification products is given in Table 2. These primer pairs amplified both mitochondrial regions in the animals of this study. Amplification of the mtDNA genes was carried out by PCR reactions of 20 *μ*L total volume, which contained about 20 ng of total DNA, 2 *μ*L Taq buffer 10×, 1 *μ*mol/L forward primer, 1 *μ*mol/L reverse primer, 3 mmol/L MgCl_2_, 10 mmol/L dNTP's, 0.7 units/*μ*L of Taq DNA polymerase (Minotech) and nano‐pure sterilized water. PCR amplification profile included an initial denaturation step at 94°C for 5 min, followed by 30 cycles of denaturation at 94°C for 1 min, annealing for 1 min, and extension at 72°C for 1 min and a final extension of 10 min at 72°C. Specificity of the PCR product was checked by electrophoresis in 1% agarose gels with mixture of 3 *μ*L of the PCR product, 3 *μ*L of agarose 6× loading dye, 4 *μ*L of nano‐pure water and molecular size marker (GeneRuler^™^ DNA Ladder Mix; Thermo Fisher Scientific, Massachusetts, USA).

**Table 2:** MtDNA primer combinations used for the amplification of the partial 16s rRNA and the cyt *b* genes. R (*rid*), L (*les*), and E (*epe*) mtDNA types

Locus	Primer pair	Forward primer(5′–3′) Reverse primer (5′–3′)	Amplified length (bp)	Species specificity	Reference
1. 16s rRNA	16Sar	CGCCTCTTGCTTAAAAACAT	609	L, R, E	Palumbi (1996)
	16Sbr	CCGGTCTGAACTCAGATCACGT			
2. 16s rRNA	16Sar	CGCCTCTTGCTTAAAAACAT	443	R, E	Forward: Palumbi (1996)
	Rid16S	TAACTTGGTTCGTTGATCAA(AC)			Reverse: this study
3. 16s rRNA	16Sar	CGCCTCTTGCTTAAAAACAT	443	L	Forward: Palumbi (1996)
	Les16S	TAACTTGGTTCGTTGATCAATT			Reverse: this study
4. cyt *b*	L14850	TCTCATCCTGATGAAACTTTGGTCC	605	L, R, E	Tanaka et al. (1996)
	H15410	GTCTTTGTAGGAGAAGTATGG			
5. cyt *b*	LCyt*b*	CTCCTGGGAGTCTGCCTAATC	579	L, R, E	Forward: this study
	H15410	GTCTTTGTAGGAGAAGTATGG			Reverse: Tanaka et al. (1996)
6. cyt *b*	RidCyt*b*	CCCAAATCGCCACAGGCC	557	R, E	Forward: this study
	H15410	GTCTTTGTAGGAGAAGTATGG			Reverse: Tanaka et al. (1996)
7. cyt *b*	LesCyt*b*	GCCCAAATCGCAACAGGTT	558	L	Forward: this study
	H15410	GTCTTTGTAGGAGAAGTATGG			Reverse: Tanaka et al. (1996)

PCR products amplified by the universal primer pairs were purified using the QIAquick PCR Purification Kit (Qiagen, Hilden, Germany) and subsequently sequenced. The sequences were obtained using the BigDyeDeoxy Terminator Cycle Sequencing method on PT100 DNA Capillary Sequencing System (MG Research). Nucleotide sequences were aligned and combined in a single data matrix by ClustalW (Thompson et al. 2002). Nucleotide ambiguities were resolved by comparing complementary strands of the same sequence. In total, DNA sequences of 459 bp in length were obtained from 77 specimens for the 16S rRNA gene, while 500 bp fragment was amplified and sequenced for the cyt *b* gene from 79 animals in our collection. Using BLAST, all mtDNA sequences from the six studied localities were assigned to species.

### Scoring of an animal's mtDNA content by restriction fragment length polymorphism (RFLP)

The type of mtDNA carried by each animal was identified by the RFLP patterns which could separate the different species. The sequences from previously amplified mtDNA genes were analyzed in the DNAMAN program that provided a restriction map and enzyme cleavage positions on the sequence. These restriction profiles allowed choosing the restriction enzymes which unambiguously identified a PCR product of each animal as belonging to a certain haplotype of this study. The 16S rRNA products were digested with the enzymes *Rsa* I and *Hae* III, while the cyt *b* products with *Hae* III and *Mbo*I, following the manufacturer's instructions (Minotech). Restriction fragments were separated by mobility after electrophoresis in 1.5% agarose gels.

### Scoring of an animal's mtDNA heteroplasmy

For detailed heteroplasmy analysis, we designed species‐specific primers for the both mitochondrial genes using previous sequence alignment. The new primers in combination with one from Palumbi (1996) or Tanaka et al. (1996) amplified either the *les* or the *rid* and the *epe* types (Table 2). Each animal from the six localities was analyzed by PCR amplification with species‐specific primers for both mitochondrial genes. The amplified mtDNA fragment was further analyzed by RFLP patterns to confirm its species assignment. We performed the DNA extraction, the PCR, and the analysis of the PCR products in separate areas for preventing contamination, while the PCR and restriction profiles for all animals were carried out in triplicates.

MtDNA amplification products from somatic tissue of 10 heteroplasmic females from Orlovat, Pancevo, Ulcinj, and Ioannina were cloned and subsequently sequenced for the heteroplasmy verification. PCR product was cloned by the pGEM^®^‐T Easy Vector System (Promega, Madison, Wisconsin, USA) according to the manufacturer's instructions. The specificity and size of inserted DNA fragment (PCR product) was examined by standard 1% agarose gel electrophoresis. A number of positive clones with inserted mtDNA fragment from either parental species were grown, amplified, and digested with restriction enzymes according to the manufacturer's instructions (Minotech) to determine mtDNA species assignment. Desired colonies were grown overnight in LB buffer, and isolation of recombinant plasmid DNA from bacteria was carried out by NucleoSpin^**®**^ Plasmid Kit. Yields and integrity of plasmid DNA for direct sequencing was carried out by a standard 1% agarose gel electrophoresis.

### Genetic distances and phylogenetic trees

Genetic distances among haplotypes were estimated using the Kimura's two‐parameter method (Kimura 1981). Phylogenetic reconstruction was carried out using the maximum likelihood method as implemented by the MEGA6 program (Tamura et al. 2013). Bootstrap values were computed from 1000 replications to obtain approximate confidence levels for the tree. Additional mtDNA sequences of *P. cretensis, P. bedriagae,* and *P. saharicus* retrieved from the GenBank (Lymberakis et al. 2007) were used for the comparison with mtDNA sequences from our samples.

## Results

### Microsatellite genotyping

The microsatellite genotypes for the six microsatellite loci (Res 5, Res 14, Res 16, Res 17, Res 20, and RLCA1b5) were used for the taxonomic identification of the pure animals and their hybrids (Table 1) in combination with their mitochondrial DNA background (Fig. 2). The *rid* and *les* genomes were unambiguously distinguished by their own private alleles for the loci Res 14, Res 16, and RLCA1b5 (Christiansen 2005) and by the presence of amplification products in either species for the Res 17 and Res 20 loci. The *epe* genome shared the same alleles with the *rid* and *les* genomes for all microsatellites, except for the Res 20 locus. This locus amplified separate set of private alleles for the *les* and *epe* genomes, with especially high number of alleles for the *epe* genome in the isolated Lysimachia population in western Greece. The hybrid animals in this study had a combination of nuclear backgrounds of the *rid* alleles with either the *les* or the *epe* ones. For two more species, *P. bedriagae* and *P. cretensis*, we amplified all microsatellite loci. Both species shared many alleles with the species of this study (Table 1).

### MtDNA haplotypes in this study

**Figure 3:**
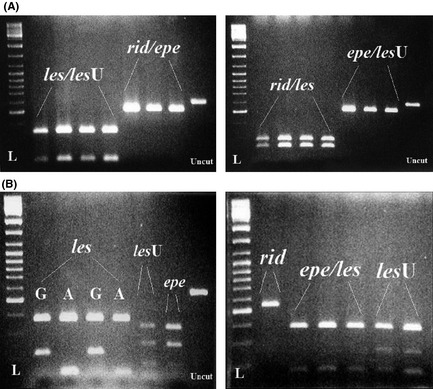
(A) Digestion patterns of the partial 16s rRNA gene of main haplotypes: *rid*,* les*,* epe,* and *les* U on agarose gel as obtained by *Rsa*I (on the left) and *Hae* III (on the right). L – ladder, uncut – undigested the 16s rRNA gene product. (B) Digestion patterns of the partial cyt *b* gene of main haplotypes: *rid*,* les*,* epe,* and *les* U on agarose gel as obtained by *Mbo*I (on the left) and *Hae* III (on the right). The *rid* haplotype has the same restriction pattern for both enzymes. L – ladder, uncut – undigested the cyt *b* gene product.

The 16S rRNA and the cyt *b* genes were amplified in all individuals by different primer pairs given in Table 2. For the 16S rRNA locus, *Rsa* I produced different restriction profile for the *les*/*les* U and the *rib*/*epe* types, while *Hae* III differentiated the *rib*/*les* from the *epe*/*les* U type (Fig. 3A). For the cyt *b* gene, the *Hae* III produced different profile for each mtDNA type, while the *Mbo* I different for the *rid*, the *epe/les* and the *les* U (Fig. 3B). In this way, every individual was classified according to whether it contained the *rid*,* epe*,* les,* or *les* U mtDNA type at both genes restriction profiles. The first three mtDNA types were similar to those previously described under the same names (Lymberakis et al. 2007), and the fourth type was detected in this study.

### Population structure based on mtDNA haplotypes and microsatellite variation

Pure *P. ridibundus* individuals were found in all studied populations, and the *rid* nuclear genome was present in all hybrid animals. The Orlovat and Pancevo populations in northern Serbia consisted of *P. ridibundus* and *P. esculentus* hybrids of both sexes that had both the *rid* and *les* nuclear background. The Nis population consisted of pure *P. ridibundus* with the *rid* nuclear background, but four individuals were triploids and characterized as *P. esculentus*. The Ulcinj population in Montenegro was a mixture of *P. ridibundus* and *P. esculentus* of both genders and the hybrids had either the *rid* or the *les* U mtDNA. The Ioannina population in Epirus, northwestern Greece, contained males of pure *P. ridibundus* and female hybrids that carried both the *rid* and *epe* nuclear genes. All males, except one, had the *rid* mtDNA, and all females, except one, had the *epe* mtDNA. We used Fisher's exact test of independence to check the gender‐mtDNA type association in this population. The hypothesis of an even chance of the *epe* or *rid* haplotypes between sexes is rejected at *P* = 0.0039, and there was a significant difference in these haplotypes proportion between females and males. The frequency of *P. ridibundus* microsatellite alleles in the Lysimachia population was much smaller compared to *P. epeiroticus*, suggesting that the introgression from *P. ridibundus* may be minor.

### Heteroplasmy analysis in surveyed populations

**Figure 4:**
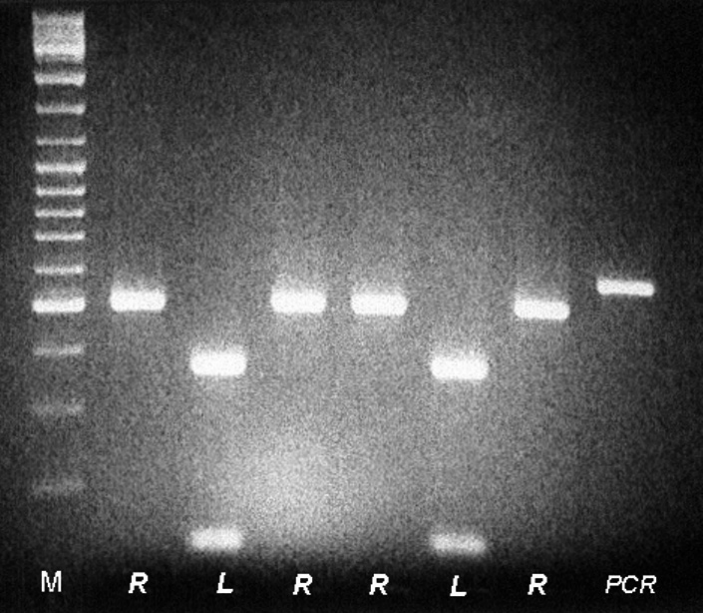
The heteroplasmy pattern for the cyt *b* gene products of seven positive clones from female hybrid from Orlovat (northern Serbia) after digestion by *Mbo*I. First line M: ladder mix, *R* – *rid*,* L* – *les*, the last line undigested PCR product.

Restriction fragment length polymorphism patterns of the analyzed mtDNA genes from the hybrid animals indicated that most of them were heteroplasmic, that is*,* the mtDNA from both parental species was present in the same individual. In pure *P. ridibundus* animals with the *rid* mtDNA type and *rid* nuclear background from the Nis population, only two individuals were found to carry both, the *rid* and the *les* mtDNA. On the contrary, *P. ridibundus* animals from Orlovat, Pancevo, Ulcinj, and Ioannina showed that along with the *rid* mtDNA type they also carried the second species mtDNA, the *les, les* U, or *epe* type. We tested all hybrid animals from the same populations using species‐specific primers combinations and found that all individuals (100%) were heteroplasmic for one or both mtDNA regions. In Figure 4, both the *rid* and *les* mtDNA amplicons from the same cyt *b* region are presented, as they were acquired and sequenced from the positive clones from a single heteroplasmic hybrid female from Orlovat.

Most individuals were found to be heteroplasmic for both mtDNA regions. However, there were the cases in which only one region was heteroplasmic. In the Orlovat, Ulcinj, and Ioannina populations in about 17% cases, the individual was heteroplasmic for the 16s rRNA region but not for the cyt *b*. The absence of the amplification at the cyt *b* region was most evident for the highly differentiated *les* U haplotype of the Ulcinj locality, which differs from the other *les* haplotypes for 33 (5.5%) nucleotide substitutions for this region. In the opposite way, in the Pancevo population heteroplasmy was not detected in 13% of the individuals for the 16s rRNA region. Absence of the second type in one of two amplified regions may have resulted from a number of reasons, such as polymorphism at the primer binding site in a certain population (Theologidis et al. 2008). However, the conservative interpretation that detection of only one type of mtDNA for one region, but not both, does not prove heteroplasmy is presented in Table 3 (numbers in parenthesis). Even if we adopt the more conservative criterion of two‐region heteroplasmy, the percentage of heteroplasmy still remains significant. In that case, the heteroplasmy in these four populations Orlovat, Pancevo, Ioannina, and Ulcinj will be reduced from 100% to 96%, 87%, 93%, and 38%, respectively (Table 3). In the Lysimachia population, homoplasmy was relatively common (33% of individuals) for the conservative scoring.

**Table 3:** Number of heteroplasmic individuals with two mtDNA types detected for both or for one of the scored genes. Alternative number of heteroplasmic individuals when two mtDNA types were detected for both genes is given in parenthesis. *Rid*,* les*, and *epe* mtDNA types, n ‐ total number of individuals in a locality

Population	mtDNA type			
	*rid* + *les*	*rid* + *epe*	n	Single region heteroplasmy
Orlovat	23 (22)	0	23	16s rRNA
Pancevo	31 (27)	0	31	cyt *b*
Nis	2 (0)	0	27	16s rRNA
Ulcinj	13 (5)	0	13	16s rRNA
Ioannina	0	30 (28)	30	16s rRNA
Lysimachia	0	22 (3)	33	16s rRNA, cyt *b*
Total	69 (54)	52 (31)	157	

### Genetic distances and the concatenated mtDNA phylogeny

The pair‐wise genetic distances were calculated based on 965 bp total sequence length for the 68 concatenated sequences of the 16s rRNA and cyt *b* genes for the six localities of this study (Table S1). The sequence variable sites for both the 16s rRNA and cyt *b* genes are listed in Figures S1 and S2, respectively. MtDNA sequences from our study were compared to that found in *P. cretensis* and *P. bedriagae* (as obtain by Lymberakis et al. (2007)), which belong to the *ridibundus*–*bedriagae* lineage. On the basis of genetic distances, four main haplotypes (sequence variants) were characterized as follows: *rid*,* epe*,* les,* and *les* U from Ulcinj locality (Montenegro) (Table S1). Excluding three sequences of *P. saharicus* that were used as out‐group (Lymberakis et al. 2007), the larger distances, ranging from 0.106 to 0.118, were for comparisons involving *lessonae* or *lessonae* U from Ulcinj versus the *ridibundus*,* bedriagae*,* cretensis,* or *epeiroticus*. Among the latter, the distances vary from 0.015 to 0.089.

Within the wider phylogeny of the genus *Pelophylax* (Fig. 2), the most basal branch was that between the *lessonae* and the *ridibundus–bedriagae* lineages, as expected. There were the two highly differentiated subclusters within the *les* lineage formed by the *les* found in Orlovat and Pancevo (northern Serbia) and the *les* U haplotypes, from Ulcinj, (Montenegro). The *ridibundus–bedriagae* group consisted of two subclusters. One included the *rid* types found in all studied localities and the types that were found in a smaller part in Thrace (Dadia), the Aegean islands (Lesvos, Astypalaia and Karpathos) and Antalya (Turkey), of the whole geographic range of different *P. bedriagae* lineages. The other included one clade with haplotypes of *P. epeiroticus* of Greece, and the other with the types found in *P. cretensis*. *P. epeiroticus* formed two clearly distinct subgroups with clear differentiation, one was found only in Ioannina and the other only in Lysimachia. Also slight differentiation occurred between *rid* types found in Pancevo and elsewhere.

## Discussion

Our analysis of mtDNA and microsatellite variation in samples from six natural populations and specimens from different collections produced two types of information. One refers to the number and types of different of mitochondrial genomes and the presence of heteroplasmy carried by any single individual. The other refers to the phylogeny of the various mitotypes found and how they are distributed in the surveyed populations. Jointly, the two types of information provide a valuable insight about the population structure, reproductive biology, and colonization history of the *Pelophylax* species complex in the Balkan Peninsula.

### Mitochondrial DNA heteroplasmy in studied *Pelophylax* populations

Western Palearctic water frogs of the genus *Pelophylax* are well known for the fact that two species may produce fertile hybrids even though their nuclear genomes have diverged beyond the degree that in other groups of animals, both invertebrate and vertebrate, is compatible with hybrid fertility (Coyne and Orr 1989; Graf and Polls‐Pelaz 1989). Rokas et al. (2003) produced a model according to which paternal mtDNA leakage is more likely to happen in species in which heterospecific mating produces fertile progeny. Under these circumstances, interspecific crosses may produce heteroplasmic hybrids and if these are fertile, even partially, they would be able to transmit their heteroplasmic state to their progeny. Currently we know of a good number of species in which heteroplasmy due to sperm leakage has been noted, but in all cases, the number of heteroplasmic individuals is small and the paternal mtDNA in these individuals accounts for no more than 10^−4^ of the individual's mtDNA content (White et al. 2008). In our study, the majority of individuals in hybrid populations were found to carry two different mtDNA genomes for the same DNA region where each could be easily recognized as belonging to one or the other of the well defined mtDNA types of *rid, les, les* U or *epe*. Both mtDNA types that were found in hybrids coincided with the species whose nuclear genomes were found in these animals. This is clear evidence that the heteroplasmy is due to hybridization between individuals that belonged to different species. In turn, this implies that leakage of paternal mtDNA must be very common, perhaps the rule, in interspecific mating of *Pelophylax* at the Balkans. The degree of heteroplasmy observed in our populations is unprecedented for species with maternal mtDNA inheritance and is comparable only to species with doubly uniparental inheritance (DUI) of mtDNA, that is, in species, all of which belong to bivalvian mollusks, in which males always inherit the paternal mtDNA along the maternal one and are, therefore, obligatory heteroplasmic (Zouros 2013).

MtDNA heteroplasmy in studied hybrid populations of *Pelophylax* may occur in two ways. One is when an egg that carries only the mtDNA of one species is fertilized by a sperm of the other species and the sperm's mtDNA is not fully expelled from the fertilized egg. This would be the relatively common case of heteroplasmy through the paternal leakage that happens during heterospecific crosses. For example, a mating between *P. lessonae* and *P. ridibundus* could introduce heteroplasmy in their hybrid offspring, which then would carry along with the *les/rid* nuclear genomes the heteroplasmic mixture of *les/rid* mitochondria. As crosses between two hybrids usually give unviable progeny due to recessive deleterious mutation (Guex et al. 2002), a hybrid female of such cross could further mate with a parental species passing on either the *rid* or *les* nuclear genome and transmitting her heteroplasmic *les/rid* mitochondria to the viable offspring. Therefore, the most common way of spreading heteroplasmy in studied hybrid population is most likely continuous backcrossing of the hybrid animals with *P. ridibundus* parental species. The other case is when a female hybrid that is itself heteroplasmic (either because paternal leakage happened in the egg from which it was produced or because the leakage happened in a female along the matrilineal line from which it descended) produces eggs that carry mtDNA of both parental types. This would be the case of heteroplasmy through the egg heteroplasmy. It was not possible to decide whether a heteroplasmy seen in any given individual was due to one or the other cause and the pervasive heteroplasmy that we have seen in these hybrid populations suggests that both mechanisms might be at work. Leakage of sperm mtDNA may be common in hybrid crosses and retention of the heteroplasmic state in hybrids may last for several generations.

Mitochondrial DNA introgression of one species within the range of another has already been observed in the water frogs of central Europe, where most hybrids of *P. esculentus* populations contain the *les* mtDNA while frequently transfer the *P. ridibundus* nuclear genome to the next generation (Plotner et al. 2008). Backcrossing of these female hybrids with the mtDNA‐nuclear mosaic eggs (who carry the *les* mitochondria and transfer the *rid* nuclear genome) with *P. ridibundus* male has resulted in introgression of the *les* mtDNA into approximately one third of *P. ridibundus* populations in that area (Plotner et al. 2008). The *les* mitochondria are more effective under hypoxic conditions than the *rid* ones, which make both larvae and adults who carry this type of mitochondria less sensitive to oxygen deficiency. In this case, the *les* mtDNA in both *P. esculentus* hybrids and *P. ridibundus* may offer an adaptive advantage through their better survival rates (Plotner et al. 2008; Hofman et al. 2012).

### MtDNA phylogeny in the surveyed region

Our survey of the mtDNA phylogeny and variation was based on the previous studies of the phylogenetic relationships and distribution of the species at the Balkans and eastern Mediterranean (Lymberakis et al. 2007; Plotner et al. 2010, 2012). Nearly, all localities across the north–south transect of the Balkans in our study had a specific mtDNA signature for the mtDNA genome that coexisted with the *rid* genome, in which the *les* and *epe* clades showed significant geographic differentiation (Fig. 2). Hofman et al. (2012) found significantly more amino acid substitutions accumulated in the *les* than in the *rid* mitochondrial lineage (131 in *les* vs. 84 in *rid* mtDNA) by obtaining the complete nucleotide sequence of *les* and *rid* mitochondrial genomes. In the *les* lineage, we detected a new clade, the *les* U found only in the in Ulcinj population in Montenegro, which is highly diverged from the other *les* haplotypes found in the southern parts of the Pannonian Basin (Orlovat and Pancevo). Genetic distances between the divergent *les* U haplotype and the other *les* haplotypes were comparable to those between any two types in the *ridibundus*–*bedriagae* lineage (Table S1). This implies that the Ulcinj population has been isolated by psychical barriers of the Dinaric Mountains for a long period of time from other *Pelophylax* populations that contained *P. lessonae* genomes, or its effective population size has been very small for random drift to have a dramatic effect on differentiation. Alternatively, the Ulcinj population may have been invaded recently by another population that carried this *les* U mtDNA type and has itself been in long isolation from other populations that carry the *les* mtDNA type. In the absence of available evidence, we assume that this *les* U mtDNA haplotype might belong to *P. shqipericus*, described in Virpazar, the Skadar Lake (Montenegro), which is in the vicinity of the Ulcinj locality. Based on phylogenetic analysis of the ND2 and ND3 mtDNA regions, Plotner et al. (2012) found similar phylogenetic pattern of *P. shqipericus* within the *les* lineage to that of the *les* U in our study.

Divergence of smaller scale occurred within the *epe* clade for the Ioannina population in Epirus and for the Lysimachia population in western Greece (Fig. 2). Both populations contain the *P. epeiroticus* species (or *epe/rid* hybrids), but must have been separated from other *Pelophylax* populations in the Balkans for a long time for this divergence to accumulate. Some degree of isolation is apparent for the Pancevo population that is almost fixed for a *rid* variant that is rare in other populations, but this degree of isolation is of a much lower extent than that between the *les* U in Ulcinj and other populations of *P. lessonae* or that between *P. epeiroticus* populations in Greece. These findings have obvious implications for wild life conservation. They point to the possibility of narrow and delegate adaptations of populations to their local environments and highlight the difficulty with which a population that goes extinction for whatever reason may be reconstituted by immigration from other populations. In a deeper historical context, our results provide information about the radiation of *Pelophylax* species in the southern Balkans, the Greek islands, and the Middle East (Fig. 2). *Pelophylax lessonae* is the most distant relative of the other species, which belong to the *P. ridibundus* species‐group. The next split happened between *P. ridibundus* and *P. epeiroticus* within the *ridibundus*–*bedriagae* lineage. *Pelophylax bedriagae* apparently sprung much later from *P. ridibundus* populations of central Balkans, about the same time that *P. cretensis* in Crete sprung from *P. epeiroticus* populations in the mainland of Greece, see also in Lymberakis et al. (2007) and Plotner et al. (2010, 2012).

### Microsatellite variation of the *Pelophylax* species in the surveyed populations

In contrast to the mtDNA differentiation, there was no clear differentiation at the six microsatellite loci, except for the most distant species of *P. lessonae* and *P. ridibundus* that belong to different lineages (Table 1). The same *P. ridibundus* alleles were found in various frequencies in all populations at all loci. Also, the same *les* alleles were found in three hybrid populations from Serbia and Montenegro that include *les* mtDNA (Orlovat and Pancevo) and the divergent mtDNA *les* U (Ulcinj). *Pelophylax epeiroticus* in the Ioannina and Lysimachia populations from in Greece appeared very different for the Res 20 locus from all others, and this was furthermore indication that the two Greek populations separated from the rest even on the basis of nuclear genes. The process of speciation within the *ridibundus*–*bedriagae* lineage has undergone the gradual divergence under the allopatric conditions that has been directly associated with isolated environments and dynamic biogeographic history of the northeastern Mediterranean since the middle Miocene (i.e., since circa 11 Mya), (Plotner et al. 2010). Differentiation in allopatry had not particularly affected their adaptive phenotypic evolution since their high similarity in morphology and the less evolved traits associated with antihybridization mechanism (e.g., species‐specific mating call), facilitate different heterospecific crosses between species of this lineage and give in general viable F1 hybrids (Plotner et al. 2010).

Hybridization between *P. ridibundus* and endemic *P. epeiroticus* in Epirus and western Greece is probably the result of secondary contacts between already divergent species which overlap in the Balkan populations, as already noticed in eastern Greece, western Anatolia and in the western Peloponnese (Hotz and Uzzell 1982). The geographically restricted *epe* genomes could have the same the role in hybridization as the *les* genomes have in the European populations as they both hybridize with *P. ridibundus* and form numerous hybrids that have higher fitness compare to the parental species (Sofianidou 1996; Hotz et al. 1999). However, the persisting primary hybridizations between *P. epeiroticus* and *P. ridibundus* of the same lineage and backcrosses of hybrids with one parental species could result in considerable nuclear gene flow and transfer of mitochondrial genomes (Plotner et al. 2008). This is in contrast to numerous hybrid populations of *P*. *esculentus* in Europe in which the long‐term hemiclonal reproduction have caused significant accumulation of deleterious mutations and low genetic variability (Hotz et al. 1999; Vorburger 2001a,b). In studied hybrid populations, the role of hybrid animals that carry both, the nuclear and mtDNA genomes from different parents could be the source of important genomic diversity. Furthermore, the periodic and limited interchange between nuclear genomes from two parental species in hybrid animals could improve genetic variability and raise chances of a small and isolating population to survive in various and changing environments.

In conclusion, in the studied *Pelophylax* hybrid populations from the southern parts of the Pannonian Basin and along a north–south transect of the Balkan Peninsula *P. ridibundus* coexists and hybridizes with *P*. *lessonae* of the *lessonae* lineage, and the endemic *P*. *epeiroticus* of the *ridibundus*–*bedriagae* lineage. Specific mtDNA signatures seen in the mtDNA genomes differentiate the studied populations at the Balkans and indicate their geographic isolation and differentiation in allopatry. We found almost universal occurrence of the mitochondrial DNA heteroplasmy in hybrid populations and provided an explanation of this phenomenon based on hybridization and the mechanism that allows leakage of paternal mtDNA in hybrids. Backcrossing of the heteroplasmic female hybrid animal with sympatric *P. ridibundus* probably introduces both mitochondrial types into this parental species, and thus, heteroplasmy is maintained in a population over generations. Hybridization between species that produces the fertile heteroplasmic hybrid progeny which carries both the nuclear and mtDNA genomes from different parents along with significant nuclear gene flow and intermittent recombinations between the parental nuclear genomes could be the source of important genomic diversity.

## Conflict of Interest

None declared.

## Supporting information


**Table S1.** Mean pairwise genetic distances (lower diagonal) with standard errors (upper diagonal) of concatenated the 16S rRNA and the cyt *b* genes, for 68 sequences of this study, and 24 sequences borrowed from NHMC (Lymberakis et al. 2007).
**Figure S1.** The variable sites of 16s rRNA gene within the four haplotype groups: *rid*,* epe les* and *les* U in the examined localities.
**Figure S2.** The variable sites of the cyt *b* gene within the four haplotype groups: *rid*,* epe, les* and *les* U in the examined localities.
