# Supplementary material for: Extensive mitochondrial heteroplasmy in hybrid water frog (Pelophylax spp.) populations from Southeast Europe
Source: Ecol Evol. 2015 Sep 28;5(20):4529–41. doi: 10.1002/ece3.1692 (PMC4670067; doi:10.1002/ece3.1692)
Supplement: Supplementary file 1 — Table S1. Mean pairwise genetic distances (lower diagonal) with standard errors (upper diagonal) of concatenated the 16S rRNA and the cyt b genes, for 68 sequences of this study, and 24 sequences borrowed from NHMC (Lymberakis et al. 2007). Figure S1. The variable sites of 16s rRNA gene within the four haplotype groups: rid, epe les and les U in the examined localities. Figure S2. The variable sites of the cyt b gene within the four haplotype groups: rid, epe, les and les U in the examined localities. [file ECE3-5-4529-s001.docx]

**Supplementary Material**

**Extensive mitochondrial heteroplasmy in hybrid water frog (*Pelophylax* spp.) populations from Southeast Europe**

Jelena M. Radojičić^1,2,*^, Imre Krizmanić^3^, and Eleftherios Zouros^2^

^1^, Institute of Marine Biology, Biotechnology and Aquaculture, Hellenic Centre for Marine Research, Heraklion, Greece

^2^, Department of Biology, University of Crete, Heraklion, Greece

^3^, Institute of Zoology, Faculty of Biology, University of Belgrade, Belgrade, Serbia

^*^Corresponding author:

Institute of Marine Biology, Biotechnology and Aquaculture,

Hellenic Centre for Marine Research,

71003 Heraklion, Crete, Greece

Tel: +302810337737

Fax: +302810337870

E-mail address: [jelena@hcmr.gr](mailto:jelena@hcmr.gr)

**Table S1.** Mean pairwise genetic distances (lower diagonal) with standard errors (upper diagonal) of concatenated the 16S rRNA and the cyt *b* genes, for 68 sequences of this study, and 24 sequences borrowed from NHMC ([Lymberakis et al., 2007](#_ENREF_23)). The last column gives the sequence diversity (π) for 963bp length of concatenated mtDNA genes (n – number of sequences).

| **Species** | **1** | **2** | **3** | **4** | **5** | **6** | **7** | **8** | **π** |
| --- | --- | --- | --- | --- | --- | --- | --- | --- | --- |
| **1. *P. ridibundus*** | - | 0.006 | 0.008 | 0.009 | 0.009 | 0.011 | 0.010 | 0.014 | 0.005 (33) |
| **2. *P. bedriagae*** | 0.048 | - | 0.008 | 0.009 | 0.008 | 0.012 | 0.010 | 0.014 | 0.018 (13) |
| **3. *P. cretensis*** | 0.071 | 0.077 | - | 0.009 | 0.008 | 0.012 | 0.011 | 0.014 | 0.005 (8) |
| **4. *P. epeiroticus* Lysimachia** | 0.077 | 0.089 | 0.070 | - | 0.004 | 0.011 | 0.011 | 0.013 | 0.003 (5) |
| **5. *P. epeiroticus* Ioannina** | 0.074 | 0.081 | 0.070 | 0.015 | - | 0.011 | 0.011 | 0.014 | 0.002 (12) |
| **6. *P. lessonae* Ulcinj** | 0.114 | 0.118 | 0.117 | 0.107 | 0.107 | - | 0.008 | 0.015 | 0.002 (3) |
| **7. *P. lessonae*** | 0.109 | 0.107 | 0.106 | 0.115 | 0.113 | 0.067 | - | 0.015 | 0.004 (15) |
| **8. *P. saharicus*** | 0.140 | 0.148 | 0.142 | 0.139 | 0.142 | 0.153 | 0.156 | - | 0 (3) |

Fig. S1. The variable sites of 16s rRNA gene within the four haplotype groups: *rid*, *epe* *les* and *les* U in the examined localities. The number of sequences are given in the brackets.

***P. ridibundus***

12333444

201123223

573390348

CTTAAATGG Orlovat(1),Pancevo(8),Ioannina(5)

G........ Orlovat(1),Pancevo(2),Ioannina(1)

G.......C Pancevo(3)

GA....... Pancevo(1)

A.......C Pancevo(1)

....CT... Pancevo(1)

........C Pancevo(1),Nis(1),Ioannina(8)

...G....C Ioannina(1)

..C.....C Ioannina(1)

......CA. Ulcinj(1),Lysimachia(2)

***P. epeiroticus***

1112234

120041623

6054964896

CGCCATATGG Ioannina(6)

.........C Ioannina(3)

..G......C Ioannina(1)

G......... Ioannina(1)

.....CC... Ioannina(1)

.T.TGC.CA. Lysimachia(1)

..GTGC.CA. Lysimachia(1)

...TGC.CA. Lysimachia(4)

***P. lessonae* and *P. lessonae* U**

11112222 2222222233 333444

2944470011 1155568822 445335

5135661301 2615860136 053273

CTTTTTCCTA TCACACTATG TCAAGG Orlovat(3),Pancevo(4)

A......... .......... ....C. Pancevo(3)

.......... ..CAT..... ...... Pancevo(1)

G......... .......... ...... Pancevo(1)

.......... .......... ....C. Pancevo(1)

A......... .......... ...... Pancevo(1)

A......... .......T.. .....A Pancevo(1)

..CCCCTTAG CT...GA.AA ATG... Ulcinj(1)

.CCC.CTTAG CT...GA.AA ATG... Ulcinj(1)

..CC.CTTAG CT...GA.AA ATG... Ulcinj(3)

**Fig. S2**. The variable sites of the cyt *b* gene within the four haplotype groups: *rid*, *epe,* *les* and *les* U in the examined localities. The number of sequences are given in the brackets.

***P. ridibundus***

12222223 34444444

1401345672 60034589

2580495108 05857492

CAACACCAGA GCCCCCGT Pancevo(15)

.C...AT..G T..TT... Pancevo(2)

.C....T..G T..TT... Pancevo(1),Nis(1),Ioannina(5)

.C...AT..G T..TTATA Ulcinj(1)

.C....TT.G T..TT... Ioannina(8)

TC....T..G T..TT... Ioannina(1)

.C.TGAT..G T..TT... Ioannina(1),Lysimachia(2)

.CG...T..G T..TT... Ioannina(1)

.C....T.AG TA.TT... Ioannina(1)

.C....T..G TAATT... Ioannina(1)

***P. epeiroticus***

22233444 444

1567845556 899

5770224390 958

GCAACGCTCC TAC Ioannina(10)

.......... .G. Ioannina(1)

....G..... ... Ioannina(1)

ATGG.A.CT. ..T Lysimachia(5)

ATGG.A.CT. ... Lysimachia(2)

ATGGGAACT. ... Lysimachia(1)

ATGG.A.CTG C.. Lysimachia(1)

ATGGGA.CTG ... Lysimachia(1)

***P. lessonae* and *P. lessonae* U**

111111111 2222222223 3333333344 4444444

123336888 9033457889 2224466780 0112356911 2456779

7270393347 6858101462 0363945020 3281043014 0460495

TCCATACGTT TATTCATTAG TCTCCTTTTT TACCGCTCTT CAGCGCT Orlovat(2),Pancevo(4)

.......... .......... .......... ......G... ....A.. Orlovat(2),Pancevo(2)

.......... .......... .......... .....G.... ....... Pancevo(2)

.......... .......... .......... .......... ...G... Pancevo(1)

.......... .......... ......G... .....GG... ....AA. Pancevo(1)

.......... .......... .......... ......G... ...GA.. Pancevo(1)

....G..... .......... .......... .....GG... ....A.. Pancevo(1)

CTTT.GTACC CCCCTCCCGA CTCTTC.CCC CGTTA.CTCC TGA.T.C Ulcinj(1)
